# Curcumin-based compounds in non-malignant biliary injury and cholangiocarcinoma: mechanistic insights and translational barriers

**DOI:** 10.3389/fphar.2026.1911009

**Published:** 2026-09-16

**Authors:** Zhenxuan Chen, Tao Peng

**Affiliations:** Department of Hepatobiliary Surgery, the First Affiliated Hospital of Yangtze University, the First People’s Hospital of Jingzhou, Jingzhou, China

**Keywords:** cholangiocarcinoma, curcumin, fibroinflammatory remodeling, non-malignant biliary injury, translational barriers

## Abstract

Biliary tract diseases are frequently associated with chronic cholestasis, cholangiocyte injury, inflammation, and fibrotic remodeling. Persistent injury and dysregulated repair may create a microenvironment that promotes the development of cholangiocarcinoma (CCA). CCA is an aggressive and highly heterogeneous biliary malignancy shaped by the interplay among bile acid dysregulation, inflammatory signaling, oxidative and nitrative stress, metabolic adaptation, stromal remodeling, and therapeutic resistance. Curcumin, a major bioactive polyphenol derived from Curcuma longa, and related curcumin-based compounds have been investigated in preclinical studies for their anti-inflammatory, antioxidant, antifibrotic, and antitumor properties. Available evidence suggests that native curcumin, its analogs and derivatives, and formulation-based interventions may modulate signaling pathways involved in chronic biliary injury and CCA progression, including NF-κB, Nrf2/HO-1, and TGF-β/Smad signaling. However, the therapeutic application of native curcumin is limited by poor aqueous solubility, low oral bioavailability, rapid metabolism, and inadequate tissue-specific delivery. This review critically evaluates curcumin-based interventions across non-malignant biliary injury and established CCA, with emphasis on their proposed mechanisms, delivery strategies, evidence limitations, and translational barriers. Current evidence remains predominantly preclinical, and curcumin-based interventions should therefore be regarded as experimental candidates rather than established therapies.

## 1 Introduction

Biliary tract diseases comprise a heterogeneous group of disorders with diverse etiologies, anatomical locations, and clinical outcomes. These disorders include primary sclerosing cholangitis (PSC), gallstone-related biliary obstruction and inflammation, liver fluke-associated cholangitis, congenital biliary abnormalities, and other chronic cholestatic conditions. In selected biliary disease states, persistent cholestasis, recurrent cholangiocyte injury, chronic inflammation, and periductal fibrotic remodeling may sustain cycles of tissue damage and dysregulated repair. These processes can promote oxidative and nitrative DNA damage, inflammatory amplification, and stromal activation, thereby creating a microenvironment permissive to cholangiocarcinoma (CCA) development. However, this increased susceptibility should not be interpreted as evidence that benign biliary diseases are uniformly premalignant or inevitably progress to CCA (Ehlken et al., 2017; Fabris et al., 2019a; Fabris et al., 2019b) (Figure 1).

**FIGURE 1:**
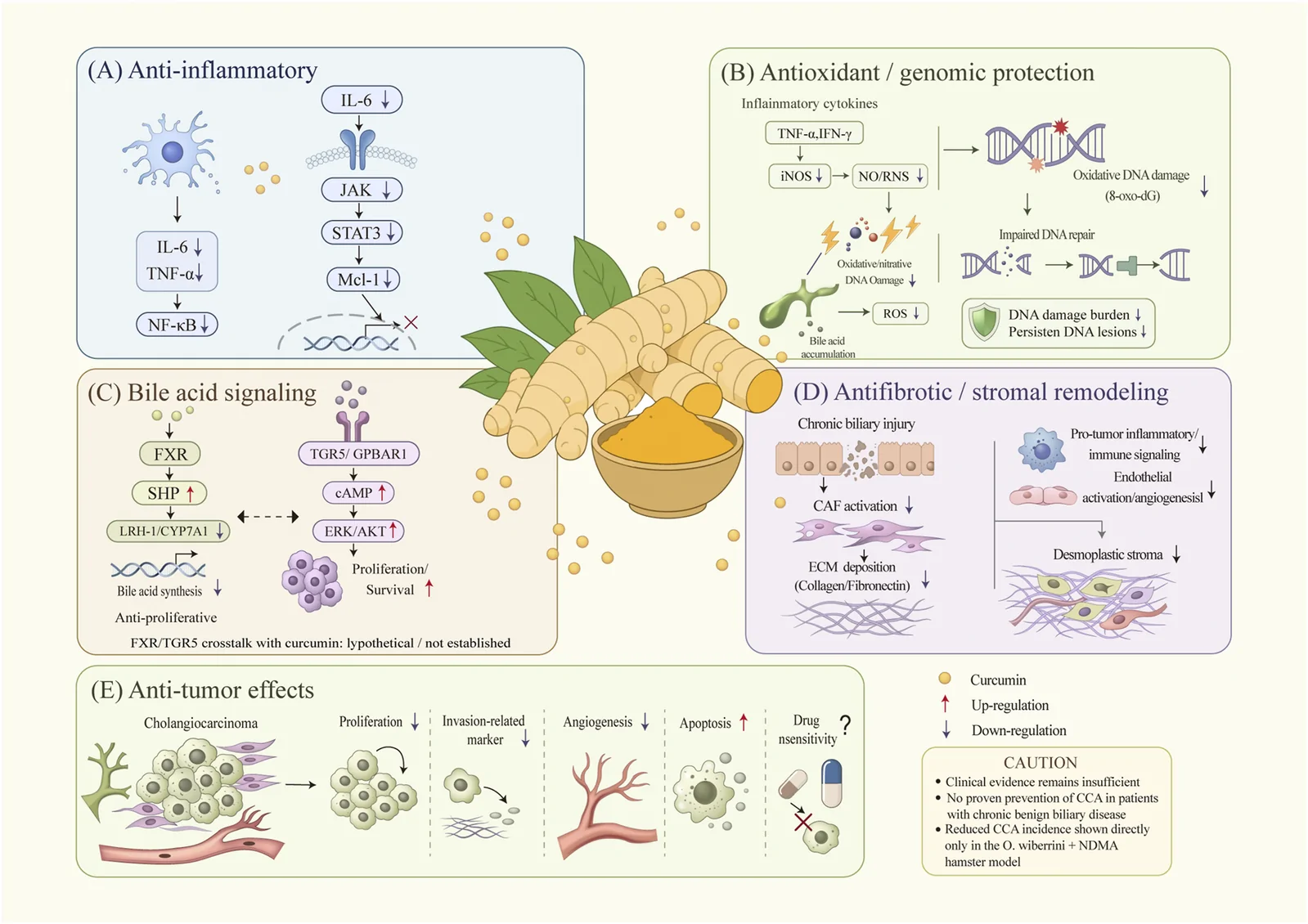
Proposed and preclinical actions of curcumin-based compounds in non-malignant biliary injury and cholangiocarcinoma: **(A)** anti-inflammatory effects; **(B)** redox regulation and genome protection; **(C)** bile acid receptor-related effects that remain unverified in cholangiocarcinoma; **(D)** antifibrotic and stromal-remodeling effects; and **(E)** antitumor effects in established cholangiocarcinoma. The scientific content was synthesized from the cited literature, and the final figure was fully redrawn by the authors using Adobe Illustrator. No third-party graphical materials were incorporated.

CCA is an aggressive biliary malignancy with marked anatomical, etiological, molecular, and microenvironmental heterogeneity. Its initiation and progression are not driven by a single molecular alteration. Instead, CCA reflects the convergence of bile acid dysregulation, chronic inflammatory signaling, oxidative and nitrative stress, fibrotic remodeling, metabolic adaptation, tumor-stromal crosstalk, immune microenvironment remodeling, and therapeutic resistance (Nakanuma and Kakuda, 2015; Gopal et al., 2024). This biological complexity may partly explain why interventions targeting a single pathway are unlikely to fully interrupt the progression from chronic biliary injury to malignant transformation or to effectively control established CCA. Multi-target compounds capable of modulating inflammatory, redox-sensitive, fibrogenic, and tumor-survival networks may therefore provide biologically plausible adjunctive strategies for non-malignant biliary injury and CCA (Vera-Ramirez et al., 2013; Cheng et al., 2022).

Curcumin is the principal bioactive polyphenol derived from the rhizome of “Curcuma longa”, and is the most extensively studied member of the curcuminoid family. In addition to native curcumin, curcumin-based compounds include naturally occurring curcuminoids, structurally optimized synthetic analogues, and formulation-based delivery systems designed to improve pharmacokinetic and tissue-targeting properties (Fu et al., 2021; Gao et al., 2024). Preclinical studies suggest that native curcumin and related intervention classes can modulate signaling pathways involved in chronic biliary injury and CCA progression, particularly NF-κB, Nrf2/HO-1, and TGF-β/Smad (Hu et al., 2017).

Native curcumin is limited by poor aqueous solubility, low oral bioavailability, rapid metabolism, chemical instability, and insufficient tissue-specific delivery (Abd El-Hack et al., 2021). Although nanocarriers, bile acid-modified delivery systems, proniosomal formulations, and synthetic analogues may improve potency or delivery efficiency, most supporting evidence remains confined to cell-based experiments and animal models (Kan et al., 2025; Crețu et al., 2026; Thongpon et al., 2025; Adeluola et al., 2021). Moreover, CCA heterogeneity across intrahepatic, perihilar, and distal subtypes may influence drug exposure, stromal penetration, tumor uptake, and therapeutic response (Banales et al., 2020; Ilyas et al., 2023). However, curcumin-based compounds should currently be considered mechanistically promising but clinically unproven. To facilitate consistent terminology throughout this review, the operational definitions of native curcumin, natural curcuminoids and extracts, synthetic analogues, and formulation-based delivery systems are summarized in Table 1. This review examines non-malignant biliary injury as a disease context, evaluates curcumin-related interventions in established CCA, and compares current development strategies and barriers to clinical translation.

**TABLE 1:** Operational terminology for curcumin-based agents and formulations discussed in this review.

Category	Definition	Representative examples	Main limitation
Native curcumin	The parent curcumin molecule (diferuloylmethane)	Curcumin (CUR)	Poor aqueous solubility and low systemic bioavailability
Natural curcuminoids and extracts	Naturally occurring curcuminoids and standardized or crude turmeric-derived extracts	Demethoxycurcumin, bisdemethoxycurcumin, curcuminoid mixtures, turmeric extracts	Variable composition, purity, and standardization
Synthetic curcumin analogues	Chemically modified derivatives of the curcumin scaffold	EF24, WZ26, GO-Y030	Often higher *in vitro* potency, but limited pharmacokinetic, toxicological, and clinical evidence
Curcumin formulations	Delivery systems designed to improve solubility, stability, absorption, or tissue exposure	BCM-95, meriva, theracurmin, liposomal curcumin, curcumin-loaded proniosomes	Formulation-specific evidence with limited comparative and clinical validation

Native curcumin, natural curcuminoids or extracts, synthetic analogues, and formulation-based products are not pharmacologically interchangeable. Formulations represent a delivery category and may contain native curcumin, curcuminoid mixtures, or synthetic derivatives. Improvements *in vitro* potency, solubility, encapsulation efficiency, or systemic exposure do not necessarily indicate superior tumor delivery or clinical efficacy.

## 2 Curcumin in non-malignant biliary injury

### 2.1 Cholestatic and bile duct injury

Disruption of bile acid homeostasis is a central feature of cholestatic liver injury. Intrahepatic cholestasis may result from impaired hepatocellular bile formation or dysfunction of canalicular transport systems, particularly the bile salt export pump (BSEP), leading to the retention of bile acids and other biliary constituents within the liver (Halilbasic et al., 2013; Tátrai et al., 2022; Saran and Brouwer, 2023). By contrast, extrahepatic cholestasis generally results from mechanical obstruction of the extrahepatic biliary tract. The accumulation of potentially cytotoxic bile acids can promote membrane injury, mitochondrial and endoplasmic reticulum stress, inflammatory signaling, and immune-cell recruitment, thereby contributing to hepatocellular and cholangiocyte injury (Cai and Boyer, 2021; Perez and Briz, 2009). These mechanisms provide a rationale for investigating the potential hepatobiliary protective effects of curcumin. The broader links between cholestatic injury and the development of a pro-tumorigenic biliary microenvironment are discussed in Section 3.

Bile toxicity is partly governed by the balance between bile acids and biliary phospholipids. ABCB4/MDR3 mediates canalicular phosphatidylcholine secretion, and its loss of function lowers the phospholipid-to-bile acid ratio and increases hepatocyte and cholangiocyte injury (Stättermayer et al., 2020). In a single *in vitro* study, 1 μM native curcumin restored membrane localization, protein maturation, and bile salt-dependent phospholipid efflux for the endoplasmic-reticulum-retained G228 R and A934 T variants, whereas G68 R and D459H remained unresponsive (Delaunay et al., 2016; Gordo-Gilart et al., 2016). These findings suggest a mutation-specific rescue effect, although the evidence remains limited to *in vitro* models and has not been independently reproduced. Whether clinically feasible dosing can achieve and sustain sufficient unbound intracellular concentrations in human hepatocytes to reproduce this rescue effect *in vivo* remains unknown.

Bile acid-induced apoptosis represents another potential target of curcumin. Glycochenodeoxycholic acid (GCDCA), a hydrophobic bile acid that accumulates during cholestasis, promotes hepatocyte apoptosis through AP-1 associated signaling. Native curcumin dose-dependently reduced GCDCA-induced caspase-3/7 activation, supporting a protective effect against bile acid-induced cellular injury (Schmucker et al., 1990; Bernt et al., 2006).

Curcumin may also attenuate intestinal barrier dysfunction associated with biliary obstruction. In a rat model of obstructive jaundice, curcumin reduced bacterial translocation (Karatepe et al., 2010). Studies using methotrexate-induced enteritis and lipopolysaccharide-stimulated intestinal epithelial cells further suggest that suppression of NF-κB associated inflammation may contribute to this effect, although these models provide only indirect support in the context of cholestasis (Song et al., 2010). Overall, the available evidence derives from hepatocyte-based, transporter-focused, intestinal barrier, and whole-animal studies and does not provide consistent or direct evidence of cholangiocyte protection during human cholestasis.

### 2.2 Sclerosing cholangitis and cholangiocyte inflammation

Primary sclerosing cholangitis (PSC) is a chronic cholestatic disease characterized by persistent bile duct inflammation, pathological repair, and progressive periductal fibrosis, which may ultimately lead to cirrhosis and liver failure (Dyson et al., 2018; Banales et al., 2019). Activated cholangiocytes contribute to this process by expressing adhesion molecules such as vascular cell adhesion molecule-1 (VCAM-1) and interacting with immune and fibrogenic cells. Sustained activation of NF-κB, ERK, and TGF-β/Smad signaling further promotes inflammatory cell retention, fibrogenic cell activation, and extracellular matrix deposition (Afford et al., 2014; Foglia et al., 2019; Ceccherini et al., 2020).

In the Mdr2^−/−^ mouse model of PSC-like cholangiopathy, native curcumin attenuated liver injury, cholestasis, ductular reaction, and periductal fibrosis. These effects were associated with activation of cholangiocyte PPARγ signaling, reduced VCAM-1 expression, and inhibition of fibrogenic cell proliferation and ERK1/2 activation (Baghdasaryan et al., 2010). These findings suggest that curcumin may reduce PSC-like inflammation and fibrosis by limiting cholangiocyte-mediated inflammatory recruitment and fibrogenic signaling.

Additional experimental studies support a broader role for curcumin in regulating biliary fibrosis. PPARγ contributes to maintenance of the quiescent phenotype of hepatic stellate cells, whereas its downregulation is associated with NF-κB and ERK activation and acquisition of a fibrogenic phenotype (Foglia et al., 2019; Fa et al., 2017). Curcumin enhanced PPARγ-related signaling and reduced the expression of α-smooth muscle actin, connective tissue growth factor, type I collagen, and other markers of hepatic stellate cell activation (Kawahara et al., 2022). It also attenuated TGF-β1-induced EMT-like changes in cholangiocytes through modulation of CD109, Smad7, Smad2/3 phosphorylation, and Hedgehog signaling (Fan et al., 2019). 

The available clinical study did not reproduce the benefit observed in the Mdr2^−/−^ model. In a single-arm, open-label pilot study, 15 patients with PSC received the bioavailability-enhanced curcumin formulation BCM-95 at 750 mg twice daily for 12 weeks. Treatment was well tolerated, but no significant improvements were observed in alkaline phosphatase, other biochemical markers, pruritus, fatigue, or self-reported overall health (Eaton et al., 2019). Several explanations are possible, although they were not directly tested. The 12-week treatment period may have been insufficient, the selected dose may not have achieved adequate exposure, and enrollment based on alkaline phosphatase may not have identified patients most likely to respond. Alternatively, curcumin may have limited biological efficacy in human PSC. Given the small sample size, absence of a control group, and spontaneous variability in alkaline phosphatase, the study could not distinguish among these possibilities and provided no evidence of biochemical or symptomatic benefit at the tested dose and duration.

### 2.3 Pathogen-associated injury and fibrosis

Pathogen-associated biliary fibrosis is most commonly associated with infection by the liver flukes Clonorchis sinensis and Opisthorchis viverrini (Prueksapanich et al., 2018; Pengput and Schwartz, 2020). Chronic residence of these parasites within the bile ducts can induce epithelial injury, persistent inflammation, and periductal fibrosis through mechanical irritation, excretory-secretory products (ESPs), and host immune responses, thereby increasing the risk of CCA (Ceci et al., 2022). *In vitro*, *C. sinensis* ESPs stimulated non-malignant cholangiocytes and hepatic stellate cells to produce TGF-β1 and IL-6 and activate inflammatory and fibrogenic signaling (Yi et al., 2024).

In O. viverrini-infected hamsters, dietary turmeric reduced periductal inflammatory cell infiltration and improved biochemical markers of hepatobiliary injury (Boonjaraspinyo et al., 2009). Because turmeric contains multiple constituents, these effects cannot be attributed specifically to curcumin. In separate studies, 1% dietary native curcumin reduced the accumulation of 8-oxodG and 8-nitroguanine in cholangiocytes and inflammatory cells, accompanied by downregulation of iNOS, NF-κB, and COX-2 and enhancement of antioxidant defenses (Pinlaor et al., 2004; Caligiuri et al., 2023; Pinlaor et al., 2009), consistent with attenuation of inflammation-associated oxidative and nitrative DNA damage rather than nonspecific free-radical scavenging.

The antifibrotic effects of curcumin appeared to depend on treatment duration. Although only limited effects were observed during early infection, 3 months of continuous administration reduced periductal fibrosis in O. viverrini-infected hamsters, as reflected by decreased collagen deposition, α-SMA expression, hepatic hydroxyproline content, and collagen I/III mRNA levels, together with reduced TIMP-1 and TIMP-2 expression and enhanced extracellular matrix degradation capacity (Pinlaor et al., 2010). These findings are consistent with a gradual effect on persistent inflammation and fibrotic remodeling.

In a rat model of *C. sinensis* infection, curcumin combined with praziquantel (PZQ) reduced parasite viability *in vitro* and decreased worm burden, hepatobiliary injury, and fibrosis *in vivo* compared with the respective experimental controls (Lee et al., 2024). This study supports further investigation of curcumin-PZQ combination strategies, but the evidence remains preclinical and does not establish curcumin as either a substitute for PZQ or a clinically validated adjunctive treatment.

### 2.4 Gallstone-related disease

Gallstone-related biliary disorders include gallbladder and common bile duct stones, together with associated cholestasis, inflammation, and biliary obstruction. Turmeric-derived preparations and curcumin have shown choleretic, antioxidant, and anti-inflammatory effects in experimental studies, and curcumin has been reported to increase bile flow in animal models (Moga, 2003). In a high-cholesterol lithogenic model, dietary curcumin at 0.5% reduced gallstone formation and reduced biliary cholesterol concentration and the cholesterol saturation index (Srinivasan, 2017). These findings support a possible preventive or metabolic regulatory effect on cholesterol gallstone formation, but do not establish the ability to dissolve established stones in humans or relieve mechanical obstruction. Accordingly, endoscopic stone extraction, biliary drainage, antimicrobial treatment, and surgery when indicated remain the established management approaches for common bile duct stones and their complications (Gupta et al., 2024; Innes et al., 2024).

Collectively, these findings indicate that curcumin modulates several biological processes involved in chronic biliary injury, including inflammation, oxidative stress, cholestasis, and fibrotic remodeling. The available evidence across non-malignant biliary disease models is summarized in Table 2. These injury-associated processes also contribute to the development of a pro-tumorigenic biliary microenvironment, which is discussed in the following section.

**TABLE 2:** Preclinical and clinical evidence for curcumin in cholangiopathies and biliary fibrosis.

Disease/model	Curcumin intervention	Experimental model	Dose/regimen	Main findings	Key limitations	Ref
*C. sinensis* infection-associated cholangitis and fibrosis	CUR alone or combined with PZQ	*In vitro* larvae/adults; infected SD rats	12.5–100 μM for metacercariae/juveniles and 100–250 μM for adults *in vitro*; 250 mg/kg orally *in vivo*	CUR + PZQ enhanced antiparasitic activity, reduced worm burden and liver injury, and attenuated bile duct hyperplasia and periductal fibrosis, with greater histological benefits following early treatment	n = 4/group; high dose; low CUR bioavailability; CUR–PZQ pharmacokinetics were not assessed	(Lee et al., 2024)
*O. viverrini-*associated periductal fibrosis	Purified dietary CUR	Infected syrian hamsters	1% (w/w) diet for up to 6 months	Long-term CUR reduced α-SMA, collagen deposition, TIMP-1/2 and TNF-α, while promoting MMP-mediated ECM remodeling	Preventive design; high dietary dose; no significant reduction in fecal egg counts; CCA incidence was not assessed	(Pinlaor et al., 2010)
*O. viverrini-*associated oxidative DNA damage	CUR-enriched diet	Infected syrian hamsters	1% (w/w) diet for 14–90 days	Reduced 8-oxodG, 8-nitroguanine, iNOS, NF-κB and COX-2; enhanced antioxidant defenses	Surrogate endpoints; small sample size; CCA incidence was not assessed	(Pinlaor et al., 2009)
*O. viverrini*-associated cholangitis	Turmeric powder standardized to 0.25% CUR	Hamsters with *O. viverrini* infection or NDMA exposure	0.25% CUR diet for 30–60 days	Reduced periductal inflammatory-cell accumulation at 30 days and lowered ALT and direct bilirubin, although the effects were inconsistent across time points	Crude formulation; short duration; anti-inflammatory effects were inconsistent at 60 days	(Boonjaraspinyo et al., 2009)
PBC-related cholangiocyte EMT	CUR	TGF-β1-treated HIBECs; 2OA-BSA mouse model	20 μM *in vitro*; 60 mg/kg/day i.p. For 8 weeks	Inhibited EMT through TGF-β/Smad and hedgehog suppression and CD109 upregulation	n = 5/group; surrogate PBC model; possible confounding by growth inhibition	(Fan et al., 2019)
Obstructive jaundice-associated gut barrier injury	CUR	Bile duct-ligated rats	20 mg/kg/day orally for 7 days	Reduced blood-culture positivity, liver injury, TNF-α, oxidative stress and intestinal apoptotic markers	Acute model; small sample size; only blood-culture positivity was significantly reduced, with limited histological improvement	(Karatepe et al., 2010)
ANIT-induced cholestasis	CUR	WT and FXR-knockout mice	120 mg/kg/day orally for 5 days	Reduced bile acid accumulation, liver injury and inflammation through FXR-dependent signaling	High-dose pretreatment model; limited relevance to chronic human cholestasis	(Yang et al., 2016
Primary sclerosing cholangitis	Bioavailability-enhanced CUR formulation (BCM-95)	Open-label pilot trial, n = 15	750 mg twice daily for 12 weeks	Well tolerated, but biochemical and clinical outcomes were not significantly improved	Uncontrolled, single-center study; small sample; short follow-up	(Eaton et al., 2019)
PSC-like chronic cholangiopathy	Curcumin C3 complex	Mdr2^−/−^ mice; cholangiocyte and myofibroblast models	4% (w/w) diet for 4–8 weeks; 10–20 μM *in vitro*	Reduced cholestasis, inflammation and fibrosis, with PPARγ-dependent effects in cholangiocytes and ERK1/2 inhibition in portal myofibroblasts	Very high dietary dose; imperfect PSC model; limited pharmacokinetic validation	(Baghdasaryan et al., 2010)

## 3 From chronic injury to a pro-tumorigenic niche

Whereas Section 2 describes injury mechanisms in specific disease models, this section focuses on the shared biological processes that may link persistent biliary injury to malignant transformation. Chronic cholestasis and disrupted bile acid homeostasis can sustain cholangiocyte injury, while inflammatory signaling and progressive stromal remodeling may create a tissue microenvironment conducive to CCA development in susceptible disease contexts (Banales et al., 2019; Fa et al., 2017; Fuchs and Trauner, 2022) (Figure 1).

### 3.1 Cholestasis-induced oxidative and genomic injury

Cholestasis-associated bile acid accumulation can sustain oxidative stress (Stygar et al., 2026). In cultured cholangiocytes, TNF-α and IFN-γ synergistically induced iNOS/NOS2 expression, increasing nitric oxide and reactive nitrogen species. These mediators inhibited adenylyl cyclase activity and reduced cAMP-dependent fluid secretion by impairing CFTR-mediated Cl^−^ transport and AE2-mediated HCO_3_^−^ transport (Spirlì et al., 2003). Inflammation may therefore aggravate cholestasis by disrupting cholangiocyte secretory function, while retained bile acids further sustain epithelial injury.

Impaired AE2-mediated bicarbonate secretion or disruption of the apical glycocalyx weakens the biliary bicarbonate umbrella, thereby increasing cholangiocyte susceptibility to bile acid-induced injury (Hohenester et al., 2012). At the same time, sustained iNOS-derived nitric oxide can promote oxidative DNA damage and impair DNA repair, including 8-oxodG base-excision repair, thereby allowing DNA lesions to persist and potentially contribute to mutagenesis and genomic instability (Jaiswal et al., 2000; Jaiswal et al., 2001). A plausible link to carcinogenesis may therefore lie not in cholestasis alone, but in the conversion of recurrent inflammatory and secretory injury into sustained genomic stress.

### 3.2 Bile acid signaling

Bile acids also signal through the farnesoid X receptor (FXR) and G protein-coupled bile acid receptor 1 (GPBAR1/TGR5). FXR induces small heterodimer partner (SHP), which represses LRH-1-dependent CYP7A1 transcription and thereby limits bile acid synthesis (Fuchs and Trauner, 2022; Lu et al., 2000). In biliary tract cancer cells, FXR agonists have been reported to enhance sensitivity to cisplatin-induced apoptosis through SHP-dependent suppression of Bcl-xL (Wang et al., 2016). TGR5 mediates bile acid-induced cholangiocyte proliferation and protects cholangiocytes from death receptor-mediated apoptosis, illustrating how its effects may differ between physiological and malignant cellular contexts (Reich et al., 2016).

In experimental CCA models, FXR activation inhibited proliferation and migration in vitro and tumor growth in vivo, whereas TGR5 activation promoted proliferation and migration in vitro but did not significantly alter tumor growth *in vivo* (Erice et al., 2018). Curcumin has been linked to FXR or TGR5 signaling primarily in non-malignant cholestatic and metabolic models (Yang et al., 2016; Yan et al., 2018; Tian et al., 2023). However, direct evidence that either receptor mediates the antitumor effects of curcumin in CCA is currently lacking. Curcumin–bile acid receptor crosstalk should therefore be regarded as a hypothesis requiring direct experimental testing rather than an established antitumor mechanism in CCA.

### 3.3 Inflammatory signaling

Persistent IL-6 signaling can activate JAK/STAT3 and promote the growth and survival of malignant cholangiocytes. STAT3-dependent upregulation of the anti-apoptotic protein Mcl-1 contributes to resistance to cell death (Isomoto et al., 2005; Labib et al., 2019). IL-6 can also produce more durable changes in gene regulation. Chronic IL-6 exposure alters promoter methylation and the expression of growth-regulatory genes (Wehbe et al., 2006), while IL-6 mediated suppression of miR-148a and miR-152 increases DNMT1 expression and modifies the expression of methylation-dependent tumor suppressor genes (Braconi et al., 2010). In addition, methylation-mediated silencing of SOCS3 removes a key negative-feedback constraint on IL-6/STAT3 signaling, prolonging STAT3 activation and enhancing Mcl-1 expression (Isomoto et al., 2007). Together, these findings indicate that chronic inflammation may become biologically persistent through epigenetic reprogramming and disruption of signaling feedback, rather than acting only as a transient proliferative stimulus.

### 3.4 Fibrotic and stromal remodeling

Fibrotic remodeling may provide a structural, biochemical, and biomechanical context linking persistent biliary injury to malignant transformation. In a p53-deficient mouse model of chronic toxin-induced bile duct injury, p53 loss, repeated biliary injury and proliferation, and the development of a fibrotic matrix microenvironment cooperated in the formation of iCCA (Farazi et al., 2006). This model suggests the possibility that fibrotic remodeling can cooperate with genetic susceptibility and persistent tissue injury during tumor initiation. However, because it combines p53 deficiency with chemical injury, it does not establish a universal sequence applicable to all CCA etiologies or anatomical subtypes.

Established CCA is characterized by a desmoplastic stroma composed of activated fibroblasts, abundant extracellular matrix, and diverse inflammatory and immune-cell populations. Interactions among these stromal components and tumor cells can promote proliferation and invasion, modify antitumor immune responses, and contribute to treatment resistance (Ilyas et al., 2023; Vaquero et al., 2020; Ying et al., 2023; Minini and Fouassier, 2023). Thus, the p53-deficient mouse model supports a possible role for fibrotic remodeling during tumor initiation, whereas studies of established CCA more clearly support its roles in tumor progression and therapeutic resistance. The contribution of individual stromal components is likely to vary according to disease etiology, anatomical subtype, and stage.

### 3.5 Curcumin as a potential intervention

Persistent biliary inflammation, oxidative stress, and fibrotic remodeling are not only characteristic features of chronic benign biliary diseases but also important components of the pro-tumorigenic microenvironment that may facilitate the development of cholangiocarcinoma. Because curcumin exhibits anti-inflammatory, antioxidant, and antifibrotic effects in a range of preclinical systems, it has been proposed as a potential multi-target intervention capable of modifying several processes shared by chronic biliary injury and cholangiocarcinogenesis (Hao et al., 2023; Chu et al., 2024; Hernández-Aquino et al., 2020).

Evidence supporting this possibility is most apparent in experimental models of liver fluke-associated biliary disease. In Opisthorchis viverrini-infected hamsters, long-term dietary curcumin administration reduced periductal fibrosis and altered the expression of mediators involved in extracellular-matrix remodeling and inflammation (Pinlaor et al., 2010). In a related model combining O. viverrini infection with N-nitrosodimethylamine exposure, curcumin reduced cholangiocarcinoma incidence and improved survival, accompanied by suppression of inflammatory signaling, oxidative and nitrative DNA damage, cell proliferation, angiogenesis, and invasion-related markers (Prakobwong et al., 2011a). These findings suggest that curcumin may influence both the chronic injury environment and some of the subsequent processes associated with malignant progression.

Mechanistic studies further indicate that curcumin can directly affect established cholangiocarcinoma cells. In human cholangiocarcinoma cell lines, it inhibited proliferation, induced apoptosis, and suppressed NF-κB, STAT3, and Akt signaling (Prakobwong et al., 2011b). Broader non-CCA literature suggests that curcumin can modulate inflammatory and MAPK signaling (Kahkhaie et al., 2019), inflammasome activation (Hasanzadeh et al., 2020), oxidative-stress and cancer-associated pathways (Neophytou et al., 2021), and fibrogenic responses in experimental liver-fibrosis models (Chu et al., 2024; Hernández-Aquino et al., 2020). These studies provide mechanistic plausibility for a disease-modifying effect, although they should not be interpreted as direct evidence that curcumin prevents malignant transformation in patients with benign biliary disease.

Clinical translation remains uncertain. A small open-label pilot study in primary sclerosing cholangitis did not demonstrate significant improvements in cholestatic biochemical indices, symptoms, or other clinically relevant outcomes (Eaton et al., 2019). Although this study was not designed to assess cancer prevention and was limited by its small sample size and uncontrolled design, its findings underscore the gap between favorable preclinical effects and demonstrable clinical benefit.

Overall, curcumin represents a potential intervention at the interface between chronic biliary injury and cholangiocarcinoma because it targets several biological processes relevant to both disease states. Nevertheless, improvements in inflammatory signaling, oxidative-damage markers, or fibrosis should not be equated with prevention of cholangiocarcinoma. Among the evidence considered here, direct evidence of reduced cholangiocarcinoma incidence is limited to the O. viverrini/N-nitrosodimethylamine hamster model (Prakobwong et al., 2011a). Clinical evidence demonstrating that curcumin prevents cholangiocarcinoma or reduces cancer risk in patients with chronic benign biliary disease is currently lacking.

## 4 Preclinical actions in established CCA

### 4.1 Proliferation and apoptosis

CCA-cell growth and survival depend on overlapping NF-κB-, STAT3-, Notch-, and PI3K/AKT/mTOR-associated programs (Zhou et al., 2025; Bu et al., 2025; Mancarella et al., 2020; Chen and Peng, 2026; Neophytou et al., 2021; Mao et al., 2025). These networks link proliferation to metabolic adaptation, apoptosis resistance, angiogenesis, and treatment response. Most pathway studies, however, use iCCA or anatomically unclassified systems; no study directly compares matched iCCA, pCCA, and dCCA models.

Native curcumin has reduced proliferation and activated apoptotic signaling in several CCA cell systems, but the studies are not interchangeable. In KKU-series cells, it lowered cyclin D1 and c-Myc and activated caspases 8, 9, and 3 with PARP cleavage, consistent with extrinsic and mitochondrial apoptosis (Prakobwong et al., 2011b). In CCLP-1 and SG-231 cells, growth inhibition accompanied suppression of the Notch1/HES-1/survivin axis and cyclin D1, increased caspase-3/7 activity, and PARP cleavage (Koprowski et al., 2015). An ethanolic Curcuma longa extract produced similar apoptotic markers in RMCCA1 cells (Leelawat and Leelawat, 2017), but its mixed botanical composition prevents attribution to native curcumin.

All three studies were performed *in vitro*. The purified-curcumin experiments therefore require confirmation at intratumoral exposures achievable *in vivo*, while the extract study additionally requires chemical standardization. Across-study similarity in apoptotic markers does not establish equivalent potency, target engagement, or subtype response.

### 4.2 Inflammatory and redox signaling

Redox activity is dose-, time-, and context-dependent. Moderate ROS levels can support adaptive signaling and tumor-cell survival, whereas excessive ROS can overwhelm antioxidant capacity, impair mitochondrial function, and trigger cell death (Ilyas and Gores, 2013; D’Autréaux and Toledano, 2007; Zhao et al., 2023). Curcumin may therefore exert predominantly antioxidant effects under some conditions but promote oxidative stress once intracellular exposure exceeds the capacity of cellular redox defenses. This behavior is better understood as an exposure-dependent continuum than as a fixed property of the molecule.

Native curcumin illustrates exposure-dependent redox effects in CCA cells. In the reported system, antiproliferative activity was observed at relatively low concentrations and was not necessarily accompanied by measurable oxidative stress. By contrast, apoptosis at higher concentrations or longer exposure was associated with increased superoxide production, disruption of cellular redox balance, loss of mitochondrial membrane potential, reduced NF-κB activity and Bcl-xL expression, and activation of apoptotic signaling. Concurrent induction of Nrf2, upregulation of GCLC, and increased intracellular GSH indicated activation of a compensatory antioxidant response (Suphim et al., 2010). The net cellular outcome therefore appears to depend on whether the imposed oxidative burden remains within, or exceeds, the cell’s adaptive redox capacity.

Curcumin analogues intervene at different points within this network. GO-Y030 inhibited IKKβ-dependent NF-κB activation and reduced p65 nuclear translocation in HuCCT-1 and complementary non-CCA systems, whereas EF24 reduced NF-κB transcriptional and DNA-binding activity and suppressed the downstream XIAP program in CCA models (Sato et al., 2011; Yin et al., 2016). These studies support canonical NF-κB signaling as a potential vulnerability in CCA. However, GO-Y030 and EF24 have not been compared head-to-head under matched experimental conditions and should not be assumed to have equivalent potency, selectivity, target engagement, or pharmacological behavior.

EF24 and WZ26 both increased ROS, disrupted mitochondrial function, induced apoptosis, and suppressed STAT3 phosphorylation in CCA models (Bisht et al., 2019; Chen et al., 2023). Antioxidant rescue experiments support an important causal contribution of ROS to the cytotoxic effects of both compounds; notably, reversal of STAT3 inhibition by exogenous antioxidants was demonstrated explicitly for EF24. WZ26 exhibited lower short-term cytotoxicity in one normal human biliary epithelial cell line, and EF24 produced no apparent toxicity during a three-week xenograft experiment. Neither observation, however, establishes a therapeutic window. Injury to normal cholangiocytes, hepatocytes, or other tissues remains plausible if they attain comparable pro-oxidant exposure. Future dose–response studies should therefore quantify intracellular exposure, ideally including the unbound drug concentration, together with oxidative damage, mitochondrial dysfunction, target engagement, and cytotoxicity in matched malignant and non-malignant hepatobiliary models.

### 4.3 Invasion, stroma, and metastasis

CCA invasion and metastasis involve extracellular matrix degradation, altered cellular adhesion, epithelial–mesenchymal plasticity, angiogenesis, and tumor–stroma interactions (Vaquero et al., 2017). In an O. viverrini-associated hamster model, curcumin reduced tumor incidence and prolonged survival, accompanied by inhibition of NF-κB activation and reduced expression of MMP-9, ICAM-1, and VEGF (Prakobwong et al., 2011a). These changes are consistent with modulation of invasion- and angiogenesis-associated pathways in a parasite-associated chemoprevention model. However, because local invasion, circulating tumor dissemination, and metastatic burden were not directly quantified, the study does not establish an antimetastatic effect.

A proteomic study first identified S100A6, lumican, plastin-2, 14-3-3 zeta/delta, and vimentin as dysregulated during the transition from O. viverrini infection to CCA (Khoontawad et al., 2017). A subsequent intervention study showed that dietary curcumin attenuated the CCA-associated elevation of these proteins in the same parasite-associated hamster model (Khoontawad et al., 2018). S100A6, lumican, plastin-2, and 14-3-3 zeta/delta were predominantly detected in tumor cells, whereas vimentin was mainly localized to fibroblasts in periductal fibrotic tissue and tumor stroma, suggesting that curcumin may affect both malignant and stromal compartments rather than exclusively reversing tumor-cell EMT. L-plastin, also known as plastin-2 or LCP1, is an actin-bundling protein whose matrix-induced expression functionally promotes CCA cell invasion (Chaijan et al., 2014).

CCA exhibits epithelial–mesenchymal plasticity, and tumor cells may occupy partial or hybrid epithelial/mesenchymal states rather than undergoing a complete and irreversible transition (Vaquero et al., 2017; Brivio et al., 2015; Yang et al., 2020). Broader evidence from non-CCA cancer models suggests that curcuminoids can modulate EMT-related NF-κB, PI3K/AKT, TGF-β/Smad, and Wnt/β-catenin signaling, but these effects have not been systematically validated in anatomically defined CCA models (Unlu et al., 2016; Shehzad et al., 2013; Pouliquen et al., 2022).

PTEN deficiency promotes CCA exosome secretion and metastasis by impairing TFEB-mediated lysosomal biogenesis and multivesicular-body degradation. In this context, the TFEB agonist curcumin analogue C1 analogue C1 restored TFEB-dependent lysosomal activity, reduced exosome secretion, and restrained migration, invasion, and liver metastasis (Jiang et al., 2023). Current evidence is therefore concentrated in parasite-associated chemoprevention and PTEN-deficient molecular models. The available evidence is heterogeneous with respect to both experimental models and measured endpoints. The parasite-associated studies primarily address carcinogenesis and stromal remodeling, whereas the C1 study provides direct evidence of reduced metastatic behavior and liver metastasis in a PTEN-deficient molecular context. These findings support model-specific antimetastatic hypotheses but do not establish a general effect across CCA anatomical subtypes or molecular backgrounds.

### 4.4 Preliminary chemosensitization evidence

Chemoresistance is a major contributor to treatment failure in advanced CCA. Among the CCA studies reviewed here, direct evidence for curcumin-based chemosensitization is limited to anoikis-resistant or anchorage-independent cell models and one acquired gemcitabine-resistance model (San et al., 2020, Thongpon et al., 2024); observations from other cancer types cannot substitute for CCA-specific validation (Bahrami et al., 2019).

Anoikis-resistant HuCCT1 and HuH28 cells were identified, with HuCCT1 showing the clearest increases in invasive capacity and reduced gemcitabine sensitivity. In HuCCT1, these phenotypes were accompanied by increased STAT3 activation and upregulation of the anti-apoptotic proteins MCL-1 and BCL-2 (Huyen et al., 2016). In separate anchorage-independent HuCCT1 and TFK-1 models, curcumin enhanced the effects of suberoylanilide hydroxamic acid (SAHA) in both cell lines and of cisplatin in TFK-1 cells. Curcumin treatment was also associated with reductions in p-STAT3, MCL-1, ANGPTL4, and selected HDACs, including HDAC1, HDAC3, and HDAC6, although the magnitude of these changes varied according to the cell line and experimental conditions (San et al., 2020). Because these molecular changes were not causally linked to the combination effects through pathway-specific rescue experiments, they should be considered parallel candidate mechanisms rather than a validated STAT3–MCL-1–ANGPTL4 chemosensitization axis.

In an acquired gemcitabine-resistant KKU-213 B model, curcumin combined with gemcitabine enhanced antiproliferative activity, synergy according to the HSA model demonstrated at the highest tested concentration pair and additive effects at lower combinations. The combination also increased apoptosis and promoted cell accumulation in the S and G2/M phases. SLC7A8 knockdown reduced GLS and GS expression, inhibited cell growth, and increased gemcitabine sensitivity. Consistent with partial involvement of this pathway, the combination reduced LAT2 expression in resistant cells and KKU-213 B^GemR xenograft tumors (Thongpon et al., 2024). However, GLS and GS were not significantly reduced in the xenografts, indicating that the proposed downstream effects on glutamine metabolism was not consistently demonstrated *in vivo*.

The combination inhibited the growth of KKU-213 B GemR xenografts and attenuated histopathological disease features in an O. viverrini/NDMA-associated hamster model (Thongpon et al., 2024). However, only the xenograft directly modeled acquired gemcitabine resistance. The hamster experiment provides complementary evidence in an etiologically relevant model but does not establish reversal of acquired chemotherapy resistance.

Together, these studies provide preliminary evidence that curcumin may modulate survival signaling in anchorage-independent CCA cells and LAT2-associated glutamine metabolism in one acquired gemcitabine-resistant model (Huyen et al., 2016; Thongpon et al., 2024). However, these findings arise from different experimental systems and do not establish a common chemosensitization mechanism. Further validation in well-characterized CCA models, together with formal combination and pathway-specific studies, is needed before these effects can be generalized. Collectively, these studies support the biological plausibility of curcumin-based interventions in established CCA, although the available evidence remains predominantly preclinical and heterogeneous with respect to experimental models, intervention strategies, and mechanistic endpoints. A comparative summary of the current evidence is presented in Table 3.

**TABLE 3:** Antitumor effects of curcumin-based interventions in cholangiocarcinoma.

Intervention	Experimental model	Dose/regimen	Main effects and mechanisms	Key limitations	Ref
Dietary CUR	*O. viverrini*/NDMA-induced CCA in hamsters	1% (w/w) diet for up to 6 months	Reduced CCA incidence and tumor growth and improved survival; suppressed NF-κB, AP-1, STAT3, inflammation, angiogenesis and invasion, while promoting apoptosis	Preventive design; high dietary exposure; limited pharmacokinetic relevance	(Prakobwong et al., 2011a)
Dietary CUR; proteomic analysis	*O. viverrini*/NDMA-induced CCA in hamsters	1% (w/w) diet for 6 months	Reversed CCA-associated expression of S100A6, lumican, plastin-2, 14-3-3ζ/δ, vimentin, clusterin and S100A10	Exploratory associations; only three proteomic replicates per group; limited functional validation	(Khoontawad et al., 2018)
CUR	KKU-M214 CCA cells	3–30 μM	Inhibited proliferation and induced ROS-dependent mitochondrial apoptosis; modulated NF-κB/Bcl-xL, p53/Bax and Nrf2/GSH signaling	Single cell line; no *in vivo* validation; high concentrations caused necrosis	(Suphim et al., 2010)
CUR	KKU100, KKU-M156 and KKU-M214 cells	10–50 μM	Suppressed proliferation and colony formation and induced apoptosis through inhibition of NF-κB, JAK1/STAT3 and Akt and induction of PPARγ and DR4/DR5	*In vitro* only; mechanistic assays mainly used 50 μM; primary target remains unclear	(Prakobwong et al., 2011b)
CUR	CCLP-1 and SG-231 cells	0–20 μM	Reduced viability and clonogenicity and induced apoptosis through downregulation of Notch1, HES-1, survivin and cyclin D1	Two cell lines; no *in vivo* or genetic-rescue validation	(Koprowski et al., 2015)
CUR + gemcitabine	Gemcitabine-resistant CCA cells, xenografts and hamster CCA	*In vitro*: CUR 5–10 μM+ GEM 0.1–0.5 μM; xenograft: CUR 20 mg/kg/day+GEM 50 mg/kg twice weekly; hamster: CUR 50 mg/kg/day+GEM 50 mg/kg twice weekly	Enhanced antiproliferative activity, with HSA-defined synergy at the highest concentration pair; increased apoptosis and S/G2-M accumulation, with partial involvement of LAT2-associated glutamine metabolism	Predominantly one resistant cell-line model; short treatment; no pharmacokinetic interaction study	(Thongpon et al., 2024)
Curcumin-loaded proniosomes	KKU-213BGemR cells and xenografts	CUR-equivalent 5–15 μM in vitro; CMPNs 62.5 or 125 mg/kg by oral gavage three times weekly for 4 weeks (≈10 or 20 mg/kg CUR-equivalent)	Inhibited proliferation, migration and invasion and reduced xenograft growth with CHOP-associated apoptotic signaling	Inhibited proliferation, migration and invasion in vitro; 62.5 mg/kg CMPNs reduced xenograft growth and PCNA expression and increased CHOP-associated apoptotic signaling, whereas 125 mg/kg did not significantly reduce tumor volume	(Thongpon et al., 2025)
EF24	HuCCT-1, TFK-1 and HuH28 cells; xenograft and metastasis models	0.5–8 μM *in vitro;* 20 mg/kg/day i.p. *in vivo*	Inhibited proliferation, EMT, invasion and metastasis and induced G2/M arrest and apoptosis through NF-κB/XIAP suppression	Immunodeficient models; repeated i.p. Dosing; no pharmacokinetic or long-term safety data	(Yin et al., 2016)
EF24	SNU478 and HuCC-T1 cells; SNU478 xenografts	0.1–5 μM; 10 mg/kg/day i.p. for 3 weeks	Induced ROS accumulation, GSH depletion, mitochondrial dysfunction and apoptosis with ROS-dependent inhibition of STAT3 phosphorylation	Two cell lines and one xenograft model; formulation-dependent delivery; no pharmacokinetic or metastasis assessment	(Bisht et al., 2019)
WZ26	RBE, QBC-939 and HUCCA cells; QBC-939 xenografts	2.5–15 μM for functional/mechanistic assays; 5–10 mg/kg/day orally for 14 days	Inhibited growth, migration, invasion and EMT and induced ROS-dependent mitochondrial apoptosis and G2/M arrest with reduced STAT3 phosphorylation	One short-term xenograft model; direct STAT3 targeting remains undefined; no pharmacokinetic data	(Chen et al., 2023)

## 5 Development strategies and translational barriers

### 5.1 Exposure and clinical translation

Native curcumin combines poor aqueous solubility and limited intestinal permeability with chemical instability, extensive intestinal and hepatic conjugation, and rapid systemic elimination. Conventional oral dosing therefore produces low and variable circulating concentrations of the unconjugated parent compound, even at gram-level doses (Hegde et al., 2023; Naksuriya et al., 2014). Moreover, total plasma curcumin measurements may not accurately represent pharmacologically available exposure, because they may include conjugated metabolites and do not establish delivery to biliary tissue or tumor.

Human studies illustrate different aspects of this translational problem. A single oral dose of up to 12,000 mg was generally tolerated but produced minimal systemic exposure, and the single-dose design does not establish long-term safety (Lao et al., 2006). Theracurmin achieved measurable systemic exposure, but only two participants had biliary tract cancer, precluding CCA-specific conclusions (Kanai et al., 2013). As discussed in Section 2.2, a 15-patient PSC pilot study found no significant biochemical, symptomatic, or quality-of-life benefit with a bioavailability-enhanced curcuminoid formulation (Eaton et al., 2019). Although the study was small and uncontrolled, its findings indicate that use of a bioavailability-enhanced formulation alone does not ensure clinically meaningful activity in human biliary disease.

Improved solubility or plasma exposure does not necessarily indicate effective delivery to CCA tissue, because drug distribution among bile, normal hepatobiliary tissues, stroma, and tumor may differ. Future studies should therefore assess parent compound, metabolites, unbound exposure, and target engagement directly in tumor tissue or validated orthotopic models. Clinical development should proceed only when tumor exposure is pharmacologically relevant and remains tolerable to normal hepatobiliary tissues.

### 5.2 Regulatory, interaction, and long-term safety barriers

Regulatory and quality requirements vary by product type. Purified curcumin, botanical mixtures, carrier-based formulations, and synthetic analogues each require product-specific controls for composition, impurities, stability, potency, formulation characteristics, and batch consistency. Dietary-supplement status does not establish therapeutic efficacy or drug-level quality (Wu et al., 2020).

Potential drug interactions also require prospective evaluation. Curcuminoids modulate CYP enzymes and P-glycoprotein in experimental systems, but available human evidence is limited, and the magnitude and direction of clinically relevant interactions remain uncertain (Bahramsoltani et al., 2017). This issue is particularly important when curcumin formulations are combined with anticancer agents, anticoagulants, immunosuppressants, or supportive-care medications with narrow therapeutic indices. Short-term studies cannot establish long-term safety in chronic biliary disease. Future studies should assess organ toxicity, drug interactions, and formulation-specific effects, particularly in patients with impaired hepatobiliary function. High-dose nanocomplex toxicity findings should be interpreted as platform-specific rather than a class-wide safety estimate (Jantawong et al., 2021).

### 5.3 CCA-directed formulation strategies

CCA formulations should be evaluated by tumor delivery, therapeutic index, and carrier safety rather than solubility alone. Bile acid-modified systems may engage hepatobiliary transport pathways, but selective uptake by malignant cholangiocytes has not been demonstrated and preferential accumulation in normal liver remains possible. Proniosomal and nanocomplex platforms currently provide the most direct CCA-specific efficacy data.

#### 5.3.1 Curcumin-loaded maltodextrin-based proniosomes

Curcumin-loaded maltodextrin-based proniosomes (CMPNs) improved entrapment, dispersibility, stability, and *ex vivo* mucosal permeation compared with curcumin suspension (Thongpon et al., 2025). They retained activity in gemcitabine-resistant CCA cells, and the lower dose inhibited xenograft growth. However, *in vitro* activity was generally comparable to free curcumin, and the higher dose produced no additional tumor control and coincided with poorer survival. Although the small sample size prevents causal interpretation, CMPNs have not yet demonstrated selective or quantitatively confirmed tumor delivery *in vivo*.

#### 5.3.2 Nanocomplexes and other carriers

In an Opisthorchis viverrini/NDMA hamster model, oral curcumin nanocomplexes equivalent to 10 mg/kg curcumin reduced CCA incidence, biliary injury, and NF-κB and FOXM1 signaling, whereas a higher dose provided no greater benefit (Jantawong et al., 2023). Together with the CMPN findings, this suggests that dose optimization is important across formulation platforms. Six-month testing found limited toxicity at low and intermediate nanocomplex doses but reversible biochemical and histopathological abnormalities at high doses. These results cannot be extrapolated to other carriers. Polymer–lipid and PEG–cholesterol systems improve encapsulation or release but have not been evaluated for antitumor efficacy in CCA models (Wilhelm et al., 2021; Pradhan et al., 2025).

CMPNs and oral nanocomplexes currently have the strongest direct preclinical support in CCA, whereas other platforms remain less disease-validated. Clinical prioritization requires comparative tumor exposure, dose–response, and long-term safety data.

### 5.4 Analogues and combination development

Chemical modification and formulation represent distinct development routes. Formulations retain native curcumin as the active pharmaceutical ingredient, whereas analogues, conjugates, hybrids, and prodrugs are chemically modified entities with potentially different pharmacology, metabolism, distribution, and toxicity (Chelimela et al., 2024). Their pharmacokinetic and safety profiles must therefore be evaluated independently rather than inferred from conventional curcumin.

#### 5.4.1 Synthetic analogues

Among the analogues reviewed here, EF24 has the broadest CCA-specific preclinical evidence. It inhibited proliferation, migration, and invasion, induced G2/M arrest and apoptosis, and reduced xenograft growth and metastasis, with evidence implicating NF-κB/XIAP-associated signaling (Yin et al., 2016). An independent study in SNU478 and HuCC-T1 cells linked EF24 activity to ROS accumulation, GSH depletion, mitochondrial depolarization, and reduced STAT3 phosphorylation. Cyclodextrin-solubilized EF24 also suppressed xenograft growth without overt toxicity during the short study period (Bisht et al., 2019). These studies provide complementary mechanistic evidence but no clinical exposure or long-term safety data.

Other analogues have narrower and mechanistically distinct evidence. GO-Y030 inhibited IKKβ-dependent NF-κB signaling and induced apoptosis in CCA cells; its greater cellular potency than native curcumin does not establish superior pharmacokinetics or CCA-specific *in vivo* efficacy (Sato et al., 2011). WZ26 inhibited CCA-cell and xenograft growth through ROS accumulation, mitochondrial dysfunction, G2/M arrest, and STAT3 inhibition (Chen et al., 2023). C1 directly activates TFEB independently of mTOR (Song et al., 2016) and, in PTEN-deficient CCA models, restored lysosomal activity, reduced exosome release, and restrained metastatic behavior (Jiang et al., 2023). These findings support investigation of C1 in PTEN/TFEB-dysregulated CCA rather than as a general substitute for curcumin.

These analogues should be treated as separate investigational drugs. No CCA clinical evaluation has been reported for them in the literature reviewed here, and comparative data on tumor distribution, therapeutic index, off-target effects, and long-term toxicity remain lacking. Systematic head-to-head comparisons among these analogues and with standard CCA therapies are also unavailable.

#### 5.4.2 Curcumin–gemcitabine

As discussed in Section 4.4, native curcumin enhanced gemcitabine response in one acquired gemcitabine-resistant KKU-213 B GemR model, with SLC7A8/LAT2-associated glutamine metabolism proposed as a contributing mechanism. Greater antitumor activity than either monotherapy was also reported in resistant xenografts and an Opisthorchis viverrini/NDMA-associated hamster model (Thongpon et al., 2024). However, only the xenograft directly modeled acquired gemcitabine resistance, and the findings remain a single mechanistic line of evidence rather than a broadly replicated sensitization effect.

A phase I study combined Theracurmin with gemcitabine-based chemotherapy and demonstrated measurable plasma exposure without unexpected adverse events. However, only two of 16 participants had biliary tract cancer, and the study was not designed to evaluate CCA-specific efficacy, tumor exposure, or target engagement (Kanai et al., 2013). The curcumin–gemcitabine combination therefore remains preclinically supported but not clinically validated in CCA. Further development requires independent replication in molecularly and anatomically diverse models, direct tumor PK/PD assessment, and formal evaluation of dose scheduling, safety, and pharmacokinetic interactions.

### 5.5 CCA subtype heterogeneity

Current curcumin evidence relies on a limited and heterogeneous set of cell lines, KKU-derived models, parasite-associated hamster models, and predominantly subcutaneous xenografts (Thongpon et al., 2025; Prakobwong et al., 2011b; Yin et al., 2016; Bisht et al., 2019; Chen et al., 2023; Thongpon et al., 2024; Espinoza, 2023). Many models are iCCA-derived, liver-fluke-associated, or incompletely annotated with respect to anatomical subtype. Among the studies reviewed here, none directly compared curcumin, its formulations, or its analogues across matched iCCA, pCCA, and dCCA systems. The findings should therefore be interpreted as model-specific. Future studies should use a common testing framework incorporating anatomical subtype, etiology, driver alterations, stromal composition, bile-acid transporter and receptor expression, and orthotopic biodistribution. Such comparisons could distinguish genuine subtype-dependent responses from differences caused by model design or drug exposure.

## 6 Conclusion

Curcumin research in biliary disease and CCA supports biological plausibility rather than therapeutic recommendation. Native curcumin modulates inflammatory, redox, and fibrogenic pathways, while formulations may improve physicochemical or exposure characteristics, and synthetic analogues provide distinct antitumor mechanisms. However, these approaches differ in composition, pharmacology, and safety and should be evaluated separately. The absence of demonstrated clinical benefit, uncertain tumor exposure, limited subtype-specific evidence, and insufficient long-term safety data define the present evidence boundary. Future studies should prioritize anatomically and molecularly defined CCA models, direct tissue PK/PD, matched malignant and non-malignant controls, and exposure-matched comparisons of formulations and analogues. Regulatory quality, drug interactions, and chronic safety should be addressed early. Until clinical benefit is demonstrated, curcumin-based interventions should be regarded as experimental rather than clinically supported preventive or adjunctive therapies.

## References

[B1] Abd El-Hack M. E. El-Saadony M. T. Swelum A. A. Arif M. Abo Ghanima M. M. Shukry M. (2021). Curcumin, the active substance of turmeric: its effects on health and ways to improve its bioavailability. J. Sci. Food Agric. 101 (14), 5747–5762. 10.1002/jsfa.11372 34143894

[B2] Adeluola A. Zulfiker A. H. M. Brazeau D. Amin ARMR (2021). Perspectives for synthetic curcumins in chemoprevention and treatment of cancer: an update with promising analogues. Eur. J. Pharmacol. 906, 174266. 10.1016/j.ejphar.2021.174266 34146588 PMC8286352

[B3] Afford S. C. Humphreys E. H. Reid D. T. Russell C. L. Banz V. M. Oo Y. (2014). Vascular cell adhesion molecule 1 expression by biliary epithelium promotes persistence of inflammation by inhibiting effector T-cell apoptosis. Hepatology 59 (5), 1932–1943. 10.1002/hep.26965 24338559

[B4] Baghdasaryan A. Claudel T. Kosters A. Gumhold J. Silbert D. Thüringer A. (2010). Curcumin improves sclerosing cholangitis in Mdr2-/- mice by inhibition of cholangiocyte inflammatory response and portal myofibroblast proliferation. Gut 59 (4), 521–530. 10.1136/gut.2009.186528 20332524 PMC3756478

[B5] Bahrami A. Majeed M. Sahebkar A. (2019). Curcumin: a potent agent to reverse epithelial-to-mesenchymal transition. Cell Oncol. 42 (4), 405–421. 10.1007/s13402-019-00442-2 30980365 PMC12994310

[B6] Bahramsoltani R. Rahimi R. Farzaei M. H. (2017). Pharmacokinetic interactions of curcuminoids with conventional drugs: a review. J. Ethnopharmacol. 209, 1–12. 10.1016/j.jep.2017.07.022 28734960

[B7] Banales J. M. Huebert R. C. Karlsen T. Strazzabosco M. LaRusso N. F. Gores G. J. (2019). Cholangiocyte pathobiology. Nat. Rev. Gastroenterol. Hepatol. 16 (5), 269–281. 10.1038/s41575-019-0125-y 30850822 PMC6563606

[B8] Banales J. M. Marin J. J. G. Lamarca A. Rodrigues P. M. Khan S. A. Roberts L. R. (2020). Cholangiocarcinoma 2020: the next horizon in mechanisms and management. Nat. Rev. Gastroenterol. Hepatol. 17 (9), 557–588. 10.1038/s41575-020-0310-z 32606456 PMC7447603

[B9] Bernt C. Vennegeerts T. Beuers U. Rust C. (2006). The human transcription factor AP-1 is a mediator of bile acid-induced liver cell apoptosis. Biochem. Biophys. Res. Commun. 340 (3), 801–806. 10.1016/j.bbrc.2005.12.081 16380075

[B10] Bisht S. Nolting J. Wenzel J. Brossart P. Feldmann G. (2019). EF24 suppresses cholangiocellular carcinoma progression, inhibits STAT3 phosphorylation, and induces apoptosis via ROS-Mediated oxidative stress. J. Oncol. 2019, 8701824. 10.1155/2019/8701824 30949204 PMC6425401

[B11] Boonjaraspinyo S. Boonmars T. Aromdee C. Srisawangwong T. Kaewsamut B. Pinlaor S. (2009). Turmeric reduces inflammatory cells in hamster opisthorchiasis. Parasitol. Res. 105 (5), 1459–1463. 10.1007/s00436-009-1553-3 19633869

[B12] Braconi C. Huang N. Patel T. (2010). MicroRNA-dependent regulation of DNA methyltransferase-1 and tumor suppressor gene expression by interleukin-6 in human malignant cholangiocytes. Hepatology 51 (3), 881–890. 10.1002/hep.23381 20146264 PMC3902044

[B13] Brivio S. Cadamuro M. Fabris L. Strazzabosco M. (2015). Epithelial-to-mesenchymal transition and cancer invasiveness: what can we learn from cholangiocarcinoma? J. Clin. Med. 4 (12), 2028–2041. 10.3390/jcm4121958 26703747 PMC4693158

[B14] Bu Y. Chen D. Li J. Yang R. Gao J. Qu W. (2025). STAT3 induced BRD9 activation promotes intrahepatic cholangiocarcinoma progression by enhancing CD36 controlled fatty acid metabolism. Cancer Lett. 634, 218066. 10.1016/j.canlet.2025.218066 41033607

[B15] Cai S. Y. Boyer J. L. (2021). The role of bile acids in cholestatic liver injury. Ann. Transl. Med. 9 (8), 737. 10.21037/atm-20-5110 PMC8106037 33987435

[B16] Caligiuri A. Becatti M. Porro N. Borghi S. Marra F. Pastore M. (2023). Oxidative stress and redox-dependent pathways in cholangiocarcinoma. Antioxidants 13 (1), 28. 10.3390/antiox13010028 38247453 PMC10812651

[B17] Ceccherini E. Cecchettini A. Morales M. A. Rocchiccioli S. (2020). The potentiality of herbal remedies in primary sclerosing cholangitis: from in vitro to clinical studies. Front. Pharmacol. 11, 813. 10.3389/fphar.2020.00813 32587513 PMC7298067

[B18] Ceci L. Zhou T. Lenci I. Meadows V. Kennedy L. Li P. (2022). Molecular mechanisms linking risk factors to cholangiocarcinoma development. Cancers 14 (6), 1442. 10.3390/cancers14061442 35326593 PMC8945938

[B124] Chaijan S. Roytrakul S. Mutirangura A. Leelawat K. (2014). Matrigel induces L-plastin expression and promotes L-plastin-dependent invasion in human cholangiocarcinoma cells. Oncol. Lett. 8 (3), 993–1000. 10.3892/ol.2014.2239 25120647 PMC4114651

[B19] Chelimela N. Alavala R. R. Satla S. R. (2024). Curcumin - bioavailability enhancement by prodrug approach and novel formulations. Chem. Biodivers. 21 (5), e202302030. 10.1002/cbdv.202302030 38401117

[B20] Chen Z. Peng T. (2026). mTOR signaling in cholangiocarcinoma: mechanistic insights and therapeutic opportunities. Front. Pharmacol. 17, 1882709. 10.3389/fphar.2026.1882709 42528623 PMC13414117

[B21] Chen M. Qian C. Jin B. Hu C. Zhang L. Wang M. (2023). Curcumin analog WZ26 induces ROS and cell death via inhibition of STAT3 in cholangiocarcinoma. Cancer Biol. Ther. 24 (1), 2162807. 10.1080/15384047.2022.2162807 36647192 PMC9851268

[B22] Cheng C. Y. Chen C. P. Wu C. E. (2022). Precision medicine in cholangiocarcinoma: past, present, and future. Life 12 (6), 829. 10.3390/life12060829 35743860 PMC9225212

[B23] Chu Y. H. Pang B. Y. Yang M. Meng Q. Leng Y. (2024). The intervention of curcumin on rodent models of hepatic fibrosis: a systematic review and meta-analysis. PLOS One 19 (5), e0304176. 10.1371/journal.pone.0304176 38781262 PMC11115266

[B24] Crețu R. Butan S. Filimon V. Bounegru A. V. Tăbăcaru A. (2026). Curcumin delivery systems: from importance and microencapsulation techniques to molecular docking and dynamics for rational formulation design. Top. Curr. Chem. (Cham) 384 (2), 18. 10.1007/s41061-026-00553-z 42065846 PMC13135015

[B25] Delaunay J. L. Durand-Schneider A. M. Dossier C. Falguières T. Gautherot J. Davit-Spraul A. (2016). A functional classification of ABCB4 variations causing progressive familial intrahepatic cholestasis type 3. Hepatology 63 (5), 1621–1631. 10.1002/hep.28300 26474921

[B26] Dyson J. K. Beuers U. Jones D. E. J. Lohse A. W. Hudson M. (2018). Primary sclerosing cholangitis. Lancet 391 (10139), 2547–2559. 10.1016/S0140-6736(18)30300-3 29452711

[B27] D’Autréaux B. Toledano M. B. (2007). ROS as signalling molecules: mechanisms that generate specificity in ROS homeostasis. Nat. Rev. Mol. Cell Biol. 8 (10), 813–824. 10.1038/nrm2256 17848967

[B28] Eaton J. E. Nelson K. M. Gossard A. A. Carey E. J. Tabibian J. H. Lindor K. D. (2019). Efficacy and safety of curcumin in primary sclerosing cholangitis: an open label pilot study. Scand. J. Gastroenterol. 54 (5), 633–639. 10.1080/00365521.2019.1611917 31131678 PMC7895452

[B29] Ehlken H. Zenouzi R. Schramm C. (2017). Risk of cholangiocarcinoma in patients with primary sclerosing cholangitis: diagnosis and surveillance. Curr. Opin. Gastroenterol. 33 (2), 78–84. 10.1097/MOG.0000000000000335 28146445

[B30] Erice O. Labiano I. Arbelaiz A. Santos-Laso A. Munoz-Garrido P. Jimenez-Agüero R. (2018). Differential effects of FXR or TGR5 activation in cholangiocarcinoma progression. Biochim. Biophys. Acta, Mol. Basis Dis. 1864 (4 Pt B), 1335–1344. 10.1016/j.bbadis.2017.08.016 28916388

[B31] Espinoza J. L. (2023). Fluke-related cholangiocarcinoma: challenges and opportunities. Pathogens 12 (12), 1429. 10.3390/pathogens12121429 38133311 PMC10748000

[B32] Fabris L. Spirli C. Cadamuro M. Fiorotto R. Strazzabosco M. (2017). Emerging concepts in biliary repair and fibrosis. Am. J. Physiol. Gastrointest. Liver Physiol. 313 (2), G102–G116. 10.1152/ajpgi.00452.2016 28526690 PMC5582882

[B33] Fabris L. Perugorria M. J. Mertens J. Björkström N. K. Cramer T. Lleo A. (2019a). The tumour microenvironment and immune milieu of cholangiocarcinoma. Liver Int. Off. J. Int. Assoc. Study Liver 39 (1), 63–78. 10.1111/liv.14098 30907492 PMC10878127

[B34] Fabris L. Spirli C. Mertens J. (2019b). Cell interactions in biliary diseases: clues from pathophysiology and repair mechanisms to foster early assessment. Int. J. Mol. Sci. 20 (16), 3961. 10.3390/ijms20163961 31416247 PMC6719930

[B35] Fan J. Wang Q. Zhang Z. Sun L. (2019). Curcumin mitigates the epithelial-to-mesenchymal transition in biliary epithelial cells through upregulating CD109 expression. Drug Dev. Res. 80 (7), 992–999. 10.1002/ddr.21580 31403228

[B36] Farazi P. A. Zeisberg M. Glickman J. Zhang Y. Kalluri R. DePinho R. A. (2006). Chronic bile duct injury associated with fibrotic matrix microenvironment provokes cholangiocarcinoma in p53-deficient mice. Cancer Res. 66 (13), 6622–6627. 10.1158/0008-5472.CAN-05-4609 16818635

[B37] Foglia B. Cannito S. Bocca C. Parola M. Novo E. (2019). ERK pathway in activated, myofibroblast-like, hepatic stellate cells: a critical signaling crossroad sustaining liver fibrosis. Int. J. Mol. Sci. 20 (11), 2700. 10.3390/ijms20112700 31159366 PMC6600376

[B38] Fu Y. S. Chen T. H. Weng L. Huang L. Lai D. Weng C. F. (2021). Pharmacological properties and underlying mechanisms of curcumin and prospects in medicinal potential. Biomed. Pharmacother. = Biomed. Pharmacother. 141, 111888. 10.1016/j.biopha.2021.111888 34237598

[B39] Fuchs C. D. Trauner M. (2022). Role of bile acids and their receptors in gastrointestinal and hepatic pathophysiology. Nat. Rev. Gastroenterol. Hepatol. 19 (7), 432–450. 10.1038/s41575-021-00566-7 35165436

[B40] Gao T. Wang S. Zhu Z. Lin L. Luo Y. Lu M. (2024). Components from Curcuma longa (turmeric) against hepatobiliary diseases based on gut-liver axis: pharmacotherapeutic properties and potential clinical applications. Am. J. Chin. Med. 52 (2), 387–415. 10.1142/S0192415X24500162 38490808

[B41] Gopal P. Robert M. E. Zhang X. (2024). Cholangiocarcinoma: pathologic and molecular classification in the era of precision medicine. Arch. Pathol. Lab. Med. 148 (3), 359–370. 10.5858/arpa.2022-0537-RA 37327187

[B42] Gordo-Gilart R. Andueza S. Hierro L. Jara P. Alvarez L. (2016). Functional rescue of trafficking-impaired ABCB4 mutants by chemical chaperones. PLOS One 11 (2), e0150098. 10.1371/journal.pone.0150098 26900700 PMC4764328

[B43] Gupta V. Abhinav A. Vuthaluru S. Kalra S. Bhalla A. Rao A. K. (2024). The multifaceted impact of gallstones: understanding complications and management strategies. Cureus 16 (6), e62500. 10.7759/cureus.62500 39022477 PMC11252534

[B44] Halilbasic E. Claudel T. Trauner M. (2013). Bile acid transporters and regulatory nuclear receptors in the liver and beyond. J. Hepatol. 58 (1), 155–168. 10.1016/j.jhep.2012.08.002 22885388 PMC3526785

[B45] Hao M. Chu Y. Lei J. Yao Z. Wang P. Chen Z. (2023). Pharmacological mechanisms and clinical applications of curcumin: update. Aging Dis. 14 (3), 716–749. 10.14336/AD.2022.1101 37191432 PMC10187702

[B46] Hasanzadeh S. Read M. I. Bland A. R. Majeed M. Jamialahmadi T. Sahebkar A. (2020). Curcumin: an inflammasome silencer. Pharmacol. Res. 159, 104921. 10.1016/j.phrs.2020.104921 32464325

[B47] Hegde M. Girisa S. BharathwajChetty B. Vishwa R. Kunnumakkara A. B. (2023). Curcumin formulations for better bioavailability: what we learned from clinical trials thus far? ACS Omega 8 (12), 10713–10746. 10.1021/acsomega.2c07326 PMC10061533 37008131

[B48] Hernández-Aquino E. Quezada-Ramírez M. A. Silva-Olivares A. Ramos-Tovar E. Flores-Beltrán R. E. Segovia J. (2020). Curcumin downregulates smad pathways and reduces hepatic stellate cells activation in experimental fibrosis. Ann. Hepatol. 19 (5), 497–506. 10.1016/j.aohep.2020.05.006 32673649

[B49] Hohenester S. Wenniger L. M. de B. Paulusma C. C. van Vliet S. J. Jefferson D. M. Elferink R. P. O. (2012). A biliary HCO3- umbrella constitutes a protective mechanism against bile acid-induced injury in human cholangiocytes. Hepatology 55 (1), 173–183. 10.1002/hep.24691 21932391

[B50] Hu R. W. Carey E. J. Lindor K. D. Tabibian J. H. (2017). Curcumin in hepatobiliary disease: pharmacotherapeutic properties and emerging potential clinical applications. Ann. Hepatol. 16 (6), 835–841. 10.5604/01.3001.0010.5273 29055920

[B51] Huyen N. T. B. Prachayasittikul V. Chan-On W. (2016). Anoikis-resistant cholangiocarcinoma cells display aggressive characteristics and increase STAT3 activation. J. Hepato-Biliary-Pancreat Sci. 23 (7), 397–405. 10.1002/jhbp.354 27107220

[B52] Ilyas S. I. Gores G. J. (2013). Pathogenesis, diagnosis, and management of cholangiocarcinoma. Gastroenterology 145 (6), 1215–1229. 10.1053/j.gastro.2013.10.013 24140396 PMC3862291

[B53] Ilyas S. I. Affo S. Goyal L. Lamarca A. Sapisochin G. Yang J. D. (2023). Cholangiocarcinoma - novel biological insights and therapeutic strategies. Nat. Rev. Clin. Oncol. 20 (7), 471–486. 10.1038/s41571-023-00770-1 PMC10601496 37188899

[B54] Innes K. Ahmed I. Hudson J. Hernández R. Gillies K. Bruce R. (2024). Laparoscopic cholecystectomy versus conservative management for adults with uncomplicated symptomatic gallstones: the C-GALL RCT. Health Technol. Assess. 28 (26), 1–151. 10.3310/MNBY3104 PMC11228691 38943314

[B55] Isomoto H. Kobayashi S. Werneburg N. W. Bronk S. F. Guicciardi M. E. Frank D. A. (2005). Interleukin 6 upregulates myeloid cell leukemia-1 expression through a STAT3 pathway in cholangiocarcinoma cells. Hepatology 42 (6), 1329–1338. 10.1002/hep.20966 16317687

[B56] Isomoto H. Mott J. L. Kobayashi S. Werneburg N. W. Bronk S. F. Haan S. (2007). Sustained IL-6/STAT-3 signaling in cholangiocarcinoma cells due to SOCS-3 epigenetic silencing. Gastroenterology 132 (1), 384–396. 10.1053/j.gastro.2006.10.037 17241887 PMC2203612

[B57] Jaiswal M. LaRusso N. F. Burgart L. J. Gores G. J. (2000). Inflammatory cytokines induce DNA damage and inhibit DNA repair in cholangiocarcinoma cells by a nitric oxide-dependent mechanism. Cancer Res. 60 (1), 184–190. 10646872

[B58] Jaiswal M. LaRusso N. F. Shapiro R. A. Billiar T. R. Gores G. J. (2001). Nitric oxide-mediated inhibition of DNA repair potentiates oxidative DNA damage in cholangiocytes. Gastroenterology 120 (1), 191–199. 10.1053/gast.2001.20875 11208728

[B59] Jantawong C. Priprem A. Intuyod K. Pairojkul C. Pinlaor P. Waraasawapati S. (2021). Curcumin-loaded nanocomplexes: acute and chronic toxicity studies in mice and hamsters. Toxicol. Rep. 8, 1346–1357. 10.1016/j.toxrep.2021.06.021 34277359 PMC8267493

[B125] Jantawong C. Chamgramol Y. Intuyod K. Priprem A. Pairojkul C. Klungsaeng S. (2023). Curcumin-loaded nanocomplexes alleviate the progression of fluke-related cholangiocarcinoma in hamsters. Cancer Nanotech. 14, 5. 10.1186/s12645-023-00155-0

[B60] Jiang T. Y. Shi Y. Y. Cui X. W. Pan Y. F. Lin Y. K. Feng X. F. (2023). PTEN deficiency facilitates exosome secretion and metastasis in cholangiocarcinoma by impairing TFEB-Mediated lysosome biogenesis. Gastroenterology 164 (3), 424–438. 10.1053/j.gastro.2022.11.025 36436593

[B61] Kahkhaie K. R. Mirhosseini A. Aliabadi A. Mohammadi A. Mousavi M. J. Haftcheshmeh S. M. (2019). Curcumin: a modulator of inflammatory signaling pathways in the immune system. Inflammopharmacology 27 (5), 885–900. 10.1007/s10787-019-00607-3 31140036

[B62] Kan G. Chen L. Zhang W. Bian Q. Wang X. Zhong J. (2025). Recent advances in the development and application of curcumin-loaded micro/nanocarriers in food research. Adv. Colloid Interface Sci. 335, 103333. 10.1016/j.cis.2024.103333 39522421

[B63] Kanai M. Otsuka Y. Otsuka K. Sato M. Nishimura T. Mori Y. (2013). A phase I study investigating the safety and pharmacokinetics of highly bioavailable curcumin (theracurmin) in cancer patients. Cancer Chemother. Pharmacol. 71 (6), 1521–1530. 10.1007/s00280-013-2151-8 23543271

[B64] Karatepe O. Acet E. Battal M. Adas G. Kemik A. Altiok M. (2010). Effects of glutamine and curcumin on bacterial translocation in jaundiced rats. World J. Gastroenterol. 16 (34), 4313–4320. 10.3748/wjg.v16.i34.4313 20818815 PMC2937112

[B65] Kawahara A. Kanno K. Yonezawa S. Otani Y. Kobayashi T. Tazuma S. (2022). Depletion of hepatic stellate cells inhibits hepatic steatosis in mice. J. Gastroenterol. Hepatol. 37 (10), 1946–1954. 10.1111/jgh.15974 35933582

[B66] Khoontawad J. Pairojkul C. Rucksaken R. Pinlaor P. Wongkham C. Yongvanit P. (2017). Differential protein expression marks the transition from infection with Opisthorchis viverrini to cholangiocarcinoma. Mol. Cell Proteom MCP 16 (5), 911–923. 10.1074/mcp.M116.064576 28232516 PMC5417829

[B67] Khoontawad J. Intuyod K. Rucksaken R. Hongsrichan N. Pairojkul C. Pinlaor P. (2018). Discovering proteins for chemoprevention and chemotherapy by curcumin in liver fluke infection-induced bile duct cancer. PLOS One 13 (11), e0207405. 10.1371/journal.pone.0207405 30440021 PMC6237386

[B68] Koprowski S. Sokolowski K. Kunnimalaiyaan S. Gamblin T. C. Kunnimalaiyaan M. (2015). Curcumin-mediated regulation of Notch1/hairy and enhancer of split-1/survivin: molecular targeting in cholangiocarcinoma. J. Surg. Res. 198 (2), 434–440. 10.1016/j.jss.2015.03.029 25890434

[B69] Labib P. L. Goodchild G. Pereira S. P. (2019). Molecular pathogenesis of cholangiocarcinoma. BMC Cancer 19 (1), 185. 10.1186/s12885-019-5391-0 30819129 PMC6394015

[B70] Lao C. D. Ruffin M. T. Normolle D. Heath D. D. Murray S. I. Bailey J. M. (2006). Dose escalation of a curcuminoid formulation. BMC Complement. Altern. Med. 6, 10. 10.1186/1472-6882-6-10 16545122 PMC1434783

[B71] Lee S. O. Chu K. B. Yoon K. W. Heo S. I. Song J. H. Li J. (2024). Combinatorial treatment with praziquantel and curcumin reduces Clonorchis sinensis parasite burden and clonorchiasis-associated pathologies in rats. Pharmaceutics 16 (12), 1550. 10.3390/pharmaceutics16121550 PMC11678916 39771529

[B72] Leelawat S. Leelawat K. (2017). Molecular mechanisms of cholangiocarcinoma cell inhibition by medicinal plants. Oncol. Lett. 13 (2), 961–966. 10.3892/ol.2016.5488 28356985 PMC5351256

[B73] Lu T. T. Makishima M. Repa J. J. Schoonjans K. Kerr T. A. Auwerx J. (2000). Molecular basis for feedback regulation of bile acid synthesis by nuclear receptors. Mol. Cell 6 (3), 507–515. 10.1016/s1097-2765(00)00050-2 11030331

[B74] Mancarella S. Serino G. Dituri F. Cigliano A. Ribback S. Wang J. (2020). Crenigacestat, a selective NOTCH1 inhibitor, reduces intrahepatic cholangiocarcinoma progression by blocking VEGFA/DLL4/MMP13 axis. Cell Death Differ. 27 (8), 2331–2343. 10.1038/s41418-020-0505-4 32042099 PMC7370218

[B75] Mao H. Zhao X. Sun S. C. (2025). NF-κB in inflammation and cancer. Cell Mol. Immunol. 22 (8), 811–839. 10.1038/s41423-025-01310-w PMC12310982 40562870

[B76] Minini M. Fouassier L. (2023). Cancer-associated fibroblasts and extracellular matrix: therapeutical strategies for modulating the cholangiocarcinoma microenvironment. Curr. Oncol. 30 (4), 4185–4196. 10.3390/curroncol30040319 PMC10137461 37185432

[B77] Moga M. M. (2003). Alternative treatment of gallbladder disease. Med. Hypotheses 60 (1), 143–147. 10.1016/s0306-9877(02)00351-1 12450782

[B78] Nakanuma Y. Kakuda Y. (2015). Pathologic classification of cholangiocarcinoma: new concepts. Best. Pract. Res. Clin. Gastroenterol. 29 (2), 277–293. 10.1016/j.bpg.2015.02.006 25966428

[B79] Naksuriya O. Okonogi S. Schiffelers R. M. Hennink W. E. (2014). Curcumin nanoformulations: a review of pharmaceutical properties and preclinical studies and clinical data related to cancer treatment. Biomaterials 35 (10), 3365–3383. 10.1016/j.biomaterials.2013.12.090 24439402

[B80] Neophytou C. M. Trougakos I. P. Erin N. Papageorgis P. (2021). Apoptosis deregulation and the development of cancer multi-drug resistance. Cancers 13 (17), 4363. 10.3390/cancers13174363 PMC8430856 34503172

[B81] Pengput A. Schwartz D. G. (2020). Risk factors for Opisthorchis viverrini infection: a systematic review. J. Infect. Public Health 13 (9), 1265–1273. 10.1016/j.jiph.2020.05.028 32564936

[B82] Perez M. J. Briz O. (2009). Bile-acid-induced cell injury and protection. World J. Gastroenterol. 15 (14), 1677–1689. 10.3748/wjg.15.1677 19360911 PMC2668773

[B83] Pinlaor S. Ma N. Hiraku Y. Yongvanit P. Semba R. Oikawa S. (2004). Repeated infection with Opisthorchis viverrini induces accumulation of 8-nitroguanine and 8-oxo-7,8-dihydro-2’-deoxyguanine in the bile duct of hamsters via inducible nitric oxide synthase. Carcinogenesis 25 (8), 1535–1542. 10.1093/carcin/bgh157 15059927

[B84] Pinlaor S. Yongvanit P. Prakobwong S. Kaewsamut B. Khoontawad J. Pinlaor P. (2009). Curcumin reduces oxidative and nitrative DNA damage through balancing of oxidant-antioxidant status in hamsters infected with Opisthorchis viverrini. Mol. Nutr. Food Res. 53 (10), 1316–1328. 10.1002/mnfr.200800567 19753608

[B85] Pinlaor S. Prakobwong S. Hiraku Y. Pinlaor P. Laothong U. Yongvanit P. (2010). Reduction of periductal fibrosis in liver fluke-infected hamsters after long-term curcumin treatment. Eur. J. Pharmacol. 638 (1-3), 134–141. 10.1016/j.ejphar.2010.04.018 20420820

[B86] Pouliquen D. L. Boissard A. Henry C. Coqueret O. Guette C. (2022). Curcuminoids as modulators of EMT in invasive cancers: a review of molecular targets with the contribution of malignant mesothelioma studies. Front. Pharmacol. 13, 934534. 10.3389/fphar.2022.934534 PMC9304619 35873564

[B87] Pradhan A. Biswal S. Bhal S. Biswal B. K. Kundu C. N. Subuddhi U. (2025). Amphiphilic poly(ethylene glycol)-cholesterol conjugate: stable micellar formulation for efficient loading and effective intracellular delivery of curcumin. ACS Appl. Bio Mater 8 (2), 1418–1436. 10.1021/acsabm.4c01657 39907519

[B88] Prakobwong S. Khoontawad J. Yongvanit P. Pairojkul C. Hiraku Y. Sithithaworn P. (2011a). Curcumin decreases cholangiocarcinogenesis in hamsters by suppressing inflammation-mediated molecular events related to multistep carcinogenesis. Int. J. Cancer 129 (1), 88–100. 10.1002/ijc.25656 20824699

[B89] Prakobwong S. Gupta S. C. Kim J. H. Sung B. Pinlaor P. Hiraku Y. (2011b). Curcumin suppresses proliferation and induces apoptosis in human biliary cancer cells through modulation of multiple cell signaling pathways. Carcinogenesis 32 (9), 1372–1380. 10.1093/carcin/bgr032 21325634 PMC3165121

[B90] Prueksapanich P. Piyachaturawat P. Aumpansub P. Ridtitid W. Chaiteerakij R. Rerknimitr R. (2018). Liver fluke-associated biliary tract cancer. Gut Liver 12 (3), 236–245. 10.5009/gnl17102 28783896 PMC5945254

[B92] Reich M. Deutschmann K. Sommerfeld A. Klindt C. Kluge S. Kubitz R. (2016). TGR5 is essential for bile acid-dependent cholangiocyte proliferation in vivo and in vitro. Gut 65 (3), 487–501. 10.1136/gutjnl-2015-309458 26420419

[B93] Saran C. Brouwer K. L. R. (2023). Hepatic bile acid transporters and drug-induced hepatotoxicity. Toxicol. Pathol. 51 (7-8), 405–413. 10.1177/01926233231212255 37982363 PMC11014762

[B126] San T. T. Khaenam P. Prachayasittikul V. Sripa B. Kunkeaw N. Chan-On W. (2020). Curcumin enhances chemotherapeutic effects and suppresses ANGPTL4 in anoikis-resistant cholangiocarcinoma cells. Heliyon 6 (1), e03255. 10.1016/j.heliyon.2020.e03255 32051864 PMC7002900

[B94] Sato A. Kudo C. Yamakoshi H. Uehara Y. Ohori H. Ishioka C. (2011). Curcumin analog GO-Y030 is a novel inhibitor of IKKβ that suppresses NF-κB signaling and induces apoptosis. Cancer Sci. 102 (5), 1045–1051. 10.1111/j.1349-7006.2011.01886.x 21272158

[B95] Schmucker D. L. Ohta M. Kanai S. Sato Y. Kitani K. (1990). Hepatic injury induced by bile salts: correlation between biochemical and morphological events. Hepatology 12 (5), 1216–1221. 10.1002/hep.1840120523 2227821

[B96] Shehzad A. Lee J. Lee Y. S. (2013). Curcumin in various cancers. BioFactors 39 (1), 56–68. 10.1002/biof.1068 23303705

[B97] Song W. B. Wang Y. Y. Meng F. S. Zhang Q. H. Zeng J. Y. Xiao L. P. (2010). Curcumin protects intestinal mucosal barrier function of rat enteritis via activation of MKP-1 and attenuation of p38 and NF-κB activation. PLOS One 5 (9), e12969. 10.1371/journal.pone.0012969 20885979 PMC2945766

[B98] Song J. X. Sun Y. R. Peluso I. Zeng Y. Yu X. Lu J. H. (2016). A novel curcumin analog binds to and activates TFEB in vitro and in vivo independent of MTOR inhibition. Autophagy 12 (8), 1372–1389. 10.1080/15548627.2016.1179404 27172265 PMC4968239

[B99] Spirlì C. Fabris L. Duner E. Fiorotto R. Ballardini G. Roskams T. (2003). Cytokine-stimulated nitric oxide production inhibits adenylyl cyclase and cAMP-dependent secretion in cholangiocytes. Gastroenterology 124 (3), 737–753. 10.1053/gast.2003.50100 12612912

[B100] Srinivasan K. (2017). Anti-cholelithogenic potential of dietary spices and their bioactives. Crit. Rev. Food Sci. Nutr. 57 (8), 1749–1758. 10.1080/10408398.2014.1003783 26147513

[B101] Stättermayer A. F. Halilbasic E. Wrba F. Ferenci P. Trauner M. (2020). Variants in ABCB4 (MDR3) across the spectrum of cholestatic liver diseases in adults. J. Hepatol. 73 (3), 651–663. 10.1016/j.jhep.2020.04.036 32376413

[B102] Stygar D. Bogielski B. Michalczyk K. Skrzep-Poloczek B. Suchocka A. Nikiel K. (2026). Cholestatic and autoimmune liver diseases, bile duct injury, oxidative stress, and therapeutic strategies. Front. Physiol. 17, 1788973. 10.3389/fphys.2026.1788973 PMC13212037 42212255

[B103] Suphim B. Prawan A. Kukongviriyapan U. Kongpetch S. Buranrat B. Kukongviriyapan V. (2010). Redox modulation and human bile duct cancer inhibition by curcumin. Food Chem. Toxicol. Int. J. Publ. Br. Ind. Biol. Res. Assoc. 48 (8-9), 2265–2272. 10.1016/j.fct.2010.05.059 20510329

[B104] Tátrai P. Erdő F. Krajcsi P. (2022). Role of hepatocyte transporters in drug-induced liver injury (DILI)-In vitro testing. Pharmaceutics 15 (1), 29. 10.3390/pharmaceutics15010029 PMC9866820 36678658

[B105] Thongpon P. Intuyod K. Chomwong S. Pongking T. Klungsaeng S. Muisuk K. (2024). Curcumin synergistically enhances the efficacy of gemcitabine against gemcitabine-resistant cholangiocarcinoma via the targeting LAT2/glutamine pathway. Sci. Rep. 14 (1), 16059. 10.1038/s41598-024-66945-7 PMC11239878 38992159

[B106] Thongpon P. Intuyod K. Pongking T. Priprem A. Chomwong S. Tanasuka P. (2025). Curcumin-loaded maltodextrin-based proniosomes potentially effective against gemcitabine-resistant cholangiocarcinoma. ACS Appl. Bio Mater 8 (1), 913–930. 10.1021/acsabm.4c01832 39772434 PMC11752495

[B107] Tian F. Chen T. Xu W. Fan Y. Feng X. Huang Q. (2023). Curcumin compensates GLP-1 deficiency via the microbiota-bile acids axis and modulation in functional crosstalk between TGR5 and FXR in Ob/Ob mice. Mol. Nutr. Food Res. 67 (22), e2300195. 10.1002/mnfr.202300195 37712101

[B108] Unlu A. Nayir E. Dogukan Kalenderoglu M. Kirca O. Ozdogan M. (2016). Curcumin (turmeric) and cancer. J. BUON Off J. Balk. Union Oncol. 21 (5), 1051–1060. 27837604

[B109] Vaquero J. Guedj N. Clapéron A. Nguyen Ho-Bouldoires T. H. Paradis V. Fouassier L. (2017). Epithelial-mesenchymal transition in cholangiocarcinoma: from clinical evidence to regulatory networks. J. Hepatol. 66 (2), 424–441. 10.1016/j.jhep.2016.09.010 27686679

[B110] Vaquero J. Aoudjehane L. Fouassier L. (2020). Cancer-associated fibroblasts in cholangiocarcinoma. Curr. Opin. Gastroenterol. 36 (2), 63–69. 10.1097/MOG.0000000000000609 31934895

[B111] Vera-Ramirez L. Pérez-Lopez P. Varela-Lopez A. Ramirez-Tortosa M. Battino M. Quiles J. L. (2013). Curcumin and liver disease. BioFactors 39 (1), 88–100. 10.1002/biof.1057 23303639

[B112] Wang W. Zhan M. Li Q. Chen W. Chu H. Huang Q. (2016). FXR agonists enhance the sensitivity of biliary tract cancer cells to cisplatin via SHP dependent inhibition of bcl-xL expression. Oncotarget 7 (23), 34617–34629. 10.18632/oncotarget.8964 27127878 PMC5085180

[B113] Wehbe H. Henson R. Meng F. Mize-Berge J. Patel T. (2006). Interleukin-6 contributes to growth in cholangiocarcinoma cells by aberrant promoter methylation and gene expression. Cancer Res. 66 (21), 10517–10524. 10.1158/0008-5472.CAN-06-2130 17079474

[B114] Wilhelm R. K. Quirós M. I. Vargas Huertas F. Vega-Baudrit J. R. Navarro-Hoyos M. Araya-Sibaja A. M. (2021). Design of hybrid polymeric-lipid nanoparticles using curcumin as a model: preparation, characterization, and in vitro evaluation of demethoxycurcumin and bisdemethoxycurcumin-loaded nanoparticles. Polymers 13 (23), 4207. 10.3390/polym13234207 PMC8659538 34883709

[B115] Wu C. Lee S. L. Taylor C. Li J. Chan Y. M. Agarwal R. (2020). Scientific and regulatory approach to botanical drug development: a U.S. FDA perspective. J. Nat. Prod. 83 (2), 552–562. 10.1021/acs.jnatprod.9b00949 31977211

[B116] Yan C. Zhang Y. Zhang X. Aa J. Wang G. Xie Y. (2018). Curcumin regulates endogenous and exogenous metabolism via Nrf2-FXR-LXR pathway in NAFLD mice. Biomed. Pharmacother. = Biomed. Pharmacother. 105, 274–281. 10.1016/j.biopha.2018.05.135 29860219

[B117] Yang F. Tang X. Ding L. Zhou Y. Yang Q. Gong J. (2016). Curcumin protects ANIT-Induced cholestasis through signaling pathway of FXR-Regulated bile acid and inflammation. Sci. Rep. 6, 33052. 10.1038/srep33052 27624003 PMC5021964

[B118] Yang J. Antin P. Berx G. Blanpain C. Brabletz T. Bronner M. (2020). Guidelines and definitions for research on epithelial-mesenchymal transition. Nat. Rev. Mol. Cell Biol. 21 (6), 341–352. 10.1038/s41580-020-0237-9 PMC7250738 32300252

[B119] Yi J. Jeong J. H. Won J. Chung S. Pak J. H. (2024). The crosstalk between cholangiocytes and hepatic stellate cells promotes the progression of epithelial-mesenchymal transition and periductal fibrosis during Clonorchis sinensis infection. Parasites Vectors 17 (1), 151. 10.1186/s13071-024-06236-2 38519993 PMC10958959

[B120] Yin D. L. Liang Y. J. Zheng T. S. Song R. P. Wang J. B. Sun B. S. (2016). EF24 inhibits tumor growth and metastasis via suppressing NF-kappaB dependent pathways in human cholangiocarcinoma. Sci. Rep. 6, 32167. 10.1038/srep32167 27571770 PMC5004153

[B121] Ying F. Chan M. S. M. Lee T. K. W. (2023). Cancer-associated fibroblasts in hepatocellular carcinoma and cholangiocarcinoma. Cell Mol. Gastroenterol. Hepatol. 15 (4), 985–999. 10.1016/j.jcmgh.2023.01.006 PMC10040968 36708970

[B122] Zhao W. Zhuang P. Chen Y. Wu Y. Zhong M. Lun Y. (2023). “double-edged sword” effect of reactive oxygen species (ROS) in tumor development and carcinogenesis. Physiol. Res. 72 (3), 301–307. 10.33549/physiolres.935007 PMC10669002 37449744

[B123] Zhou S. Yang Y. Qi F. Sun W. Zhang D. Lu Z. (2025). ADAMDEC1 promotes the malignant progression of cholangiocarcinoma by regulating NF-κB signaling pathway. Sci. Rep. 15 (1), 817. 10.1038/s41598-025-85241-6 39755752 PMC11700112

